# Bulk-Surface Modification of Nanoparticles for Developing Highly-Crosslinked Polymer Nanocomposites

**DOI:** 10.3390/polym12081820

**Published:** 2020-08-13

**Authors:** Maryam Jouyandeh, Mohammad Reza Ganjali, Mustafa Aghazadeh, Sajjad Habibzadeh, Krzysztof Formela, Mohammad Reza Saeb

**Affiliations:** 1 Center of Excellence in Electrochemistry, School of Chemistry, College of Science, University of Tehran, Tehran 11155-4563, Iran; maryam.jouyande@gmail.com (M.J.); mustafa.aghazadeh@gmail.com (M.A.); 2 Biosensor Research Center, Endocrinology and Metabolism Molecular-Cellular Sciences Institute, Tehran University of Medical Sciences, Tehran 11155-4563, Iran; 3 Department of Chemical Engineering, Amirkabir University of Technology (Tehran Polytechnic), Tehran 1591639675, Iran; sajjad.habibzadeh@aut.ac.ir; 4 Department of Polymer Technology, Faculty of Chemistry, Gdańsk University of Technology, Gabriela Narutowicza 11/12, 80-233 Gdańsk, Poland

**Keywords:** *Cure Index*, Cure kinetics, epoxy nanocomposites, bulk modification, surface modification

## Abstract

Surface modification of nanoparticles with functional molecules has become a routine method to compensate for diffusion-controlled crosslinking of thermoset polymer composites at late stages of crosslinking, while bulk modification has not carefully been discussed. In this work, a highly-crosslinked model polymer nanocomposite based on epoxy and surface-bulk functionalized magnetic nanoparticles (MNPs) was developed. MNPs were synthesized electrochemically, and then polyethylene glycol (PEG) surface-functionalized (PEG-MNPs) and PEG-functionalized cobalt-doped (Co-PEG-MNPs) particles were developed and used in nanocomposite preparation. Various analyses including field-emission scanning electron microscopy, Fourier-transform infrared spectrophotometry (FTIR), thermogravimetric analysis (TGA), X-ray diffraction (XRD) and vibrating sample magnetometry (VSM) were employed in characterization of surface and bulk of PEG-MNPs and Co-PEG-MNPs. Epoxy nanocomposites including the aforementioned MNPs were prepared and analyzed by nonisothermal differential scanning calorimetry (DSC) to study their curing potential in epoxy/amine system. Analyses based on *Cure Index* revealed that incorporation of 0.1 wt.% of Co-PEG-MNPs into epoxy led to *Excellent* cure at all heating rates, which uncovered the assistance of bulk modification of nanoparticles to the crosslinking of model epoxy nanocomposites. Isoconversional methods revealed higher activation energy for the completely crosslinked epoxy/Co-PEG-MNPs nanocomposite compared to the neat epoxy. The kinetic model based on isoconversional methods was verified by the experimental rate of cure reaction.

## 1. Introduction

Enhancement of properties of thermoset resins via surface modification of nanoparticles has been practiced over the years [1,2,3]. Overall, a larger curing widow is the result of nanoparticle incorporation into the thermoset resins, but modification of nanoparticle surface introduced as the solution has hindered or caused incomplete crosslinking [4,5,6]. Despite such achievements, completely cured thermoset nanocomposites are rarely obtained because of dispersion and distribution of nanoparticles into the resins being difficult, mainly due to early state gelation as a consequence of using highly reactive surface-modified nanoparticles [7,8,9]. On the other hand, modification of bulk composition of nanoparticles in addition to the surface functionalization was not characteristically considered as a possible way to compensate for incomplete cure of thermoset composites. Moreover, change in the chemical interaction state in the systems containing bulk-modified particles was not effectively discussed.

**Figure 1:**
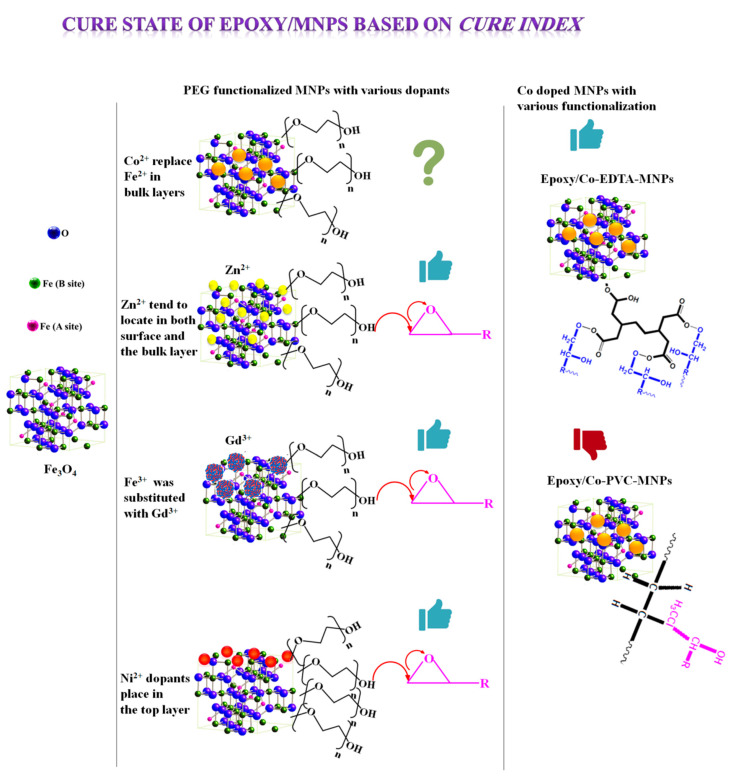
Cure state of epoxy/MNPs as model nanocomposite in which MNPs underwent various bulk and surface modifications, where 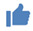 shows *Good* cure state, while 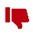 denotes *Poor* cure state.

Electrochemical methods have been practiced for years to synthesize different types of nanoparticles [10,11,12,13]. It was reported that the superparamagnetic nature of nanoparticles can be altered by doping metal cations [14,15]. In a similar manner, surface modification of magnetite nanoparticles (MNPs) with polymers has rendered MNPs exhibiting proper physicochemical characters and magnetic behavior [16,17,18,19]. In this regard, surface coating with polyethylene glycol (PEG), polyethylenimine, polyvinylpyrrolidone and polyvinyl alcohol polymers have been addressed [20,21,22]. However, the high potential of MNPs to agglomerate is known as a deterrent against epoxy network formation. It has been found that Zn^2+^ [23] dopants in the structure of Fe_3_O_4_ can position in the bulk at the surface of Fe_3_O_4_ crystals (Figure 1). When epoxy is cured with PEG functionalized Zn-doped MNPs, the *Poor* cure state was changed to *Good* cure, as measured by the *Cure Index*, which was inferred to be due to the contribution of OH groups of PEG as well as Zn^2+^ Lewis acid precursors to epoxy ring opening [24]. By contrast, the Ni^2+^ dopants mainly locate on the top layer of Fe_3_O_4_ crystal that significantly activate the MNPs surface [25]. *Good* cure state is the result of PEG functionalization of Ni^2+^-doped Fe_3_O_4_ nanoparticles [16]. The curing reaction in epoxy system similarly improved in the epoxy system containing 0.1 wt.% PEG-functionalized gadolinium (Gd)-doped Fe_3_O_4_ when Fe^3+^ was partially replaced with Gd^3+^ in the lattice [26,27]. Likewise, when Fe^2+^ was replaced with Co^2+^ in the bulk layers of Fe_3_O_4_ crystal, the cure was changed [28]. As shown in Figure 1, surface functionalization of Co-doped MNPs with different molecules posed different effects on the cure state of epoxy. For instance, the use of ethylenediaminetetraacetic acid (EDTA) as surface modifier for Co-doped MNPs makes the cure *Good* [29]. On the other hand, *Poor* cure state is obtained in the case of epoxy containing polyvinyl chloride (PVC) functionalized Co-doped MNPs [30]. Therefore, the chemistry and the position (bulk and/or surface) of dopant has a significant role in crosslinking epoxy resin.

In this work, the effect of both bulk and surface modifications of nanoparticles to cure behavior and kinetics of a model nanocomposite based on epoxy and MNPs was studied. The bare, cobalt (Co)-doped and PEG-capped MNPs were synthesized using a one-step electrochemical procedure via galvanostatic cathodic deposition (GCD). Then, the prepared nanoparticles were imaged by field-emission scanning electron microscopy (FE-SEM) for morphology assessment and Fourier-transform infrared spectrophotometry (FTIR), X-ray diffraction (XRD), vibrating sample magnetometry (VSM) and thermogravimetric analysis (TGA) for structural and surface changes. The potential of PEG-MNPs and PEG-Co-doped MNPs for being cured with epoxy was evaluated based on experimental data provided through nonisothermal differential scanning calorimetry (DSC) applied at different heating rates of 5, 10, 15 and 20 °C·min^−1^. The effects of the bulk composition and surface chemistry of nanoparticles on the curing kinetics of epoxy were considered in terms of *Cure Index*. The glass transition temperature (*T_g_*) of samples were obtained from the reheating cycle of DSC at heating rate of 10 °C·min^−1^.

## 2. Materials and Methods

FeCl_2_·4H_2_O having purity of 99.5%, Fe(NO_3_)_3_ 9H_2_O with purity of 99.9% and Co(NO_3_)_2_·4H_2_O having purity of 99.8% were purchased from Sigma-Aldrich (Paris, France) and used as received. Polyethylene glycol (PEG) having molecular weight of 1500 Da was provided by Sigma-Aldrich (France) and used as received in surface functionalization of MNPs. Diglycidyl ether of bis-phenol A, Epon-828 (DGEBA), was used as epoxy resin having epoxide equivalent weight (EEW) of 185–192 g/eq, while triethylenetetramine (TETA) was used as curing agent having hydrogen equivalent weight (HEW) of 25 g/eq, both purchased from Hexion (Beijing, China) and used in stoichiometric amount (resin:hardener ratio of 100:13) for developing nanocomposites. Dimethylformamide (DMF) was provided by Merck Chemicals Co. (Darmstadt, Germany) and used as solvent in preparation of nanocomposite dispersion.

### 2.1. Synthesis of MNPs

We synthesized two types of MNPs, i.e., PEG-functionalized and PEG-functionalized/Co-doped MNPs, through the GCD procedure, as per a well-documented recipe [31,32,33].

### 2.2. Epoxy Nanocomposite Preparation

Epoxy dispersions containing 0.1 wt.% of PEG-MNPs and Co-doped PEG-MNPs nanoparticles were prepared and denoted as EP/PEG-MNPs and EP/Co-PEG-MNPs, respectively. Dispersion was performed by the aid of a probe sonicator working under on-off duty cycle for 5 min. The pre-mixed dilute dispersion was further agitated by the aid of a mechanical mixer (2500 rpm, 20 min) keeping the stoichiometry fixed until reaching a well-mixed homogeneous liquid-like nanocomposite.

### 2.3. Characterization of Nanoparticles and Nanocomposites

FE-SEM instrument (Mira 3-XMU, Kohoutovice, Czech Republic) working under accelerating voltage of 30 kV was used to study the surface morphology of the MNPs. FTIR spectra of the untreated and bulk surface-treated MNPs were collected by the use of Bruker Vector spectrometer (22 IR, Coventry, UK) within the wavelength interval of 4000–400 cm^−1^. X-ray diffraction apparatus (PW-1800, Amsterdam, The Netherlands) with a Co Kα radiation was used to collect XRD patterns from the bulk of the synthesized MNPs. The supermagnetic response of MNPs was received in an interval of −20,000 to 20,000 Oe under ambient as complement to assessments on the structural changes by the use of a vibrational sample magnetometer (VSM, Model: lakeshore 7400, Westerville, OH, USA).

To compare the grafting percent of the PEG on the surface of MNPs and Co^2+^-doped MNPs, we used TGA by a PerkinElmer apparatus (STA-6000, Norwalk, CT, USA). The powder samples were heated in the temperature range of 25–550 °C with heating rate of 10 °C·min^−1^ under N_2_ flow circulation of 20 mL/min.

Differential scanning calorimetry (DSV) was performed on a Perkin Elmer apparatus (DSC 4000, Waltham, MA, USA) to study cure behavior and kinetics of cure reaction in epoxy and its nanocomposites. Nonisothermal scans were carried out in the temperature interval of 15–250 °C under the nitrogen flow circulation of 20 mL·min^−1^ at various heating rates (*β*): 5, 10, 15 and 20 °C·min^−1^. To obtain *T_g_* of the prepared systems, neat epoxy and its nanocomposites cured at *β* of 10 °C·min^−1^ were cooled to room temperature and again reheated to 250 °C with *β* of 10 °C·min^−1^.

## 3. Results and Discussion

### 3.1. Analysis of the Bulk and Surface of MNPs

**Figure 2:**
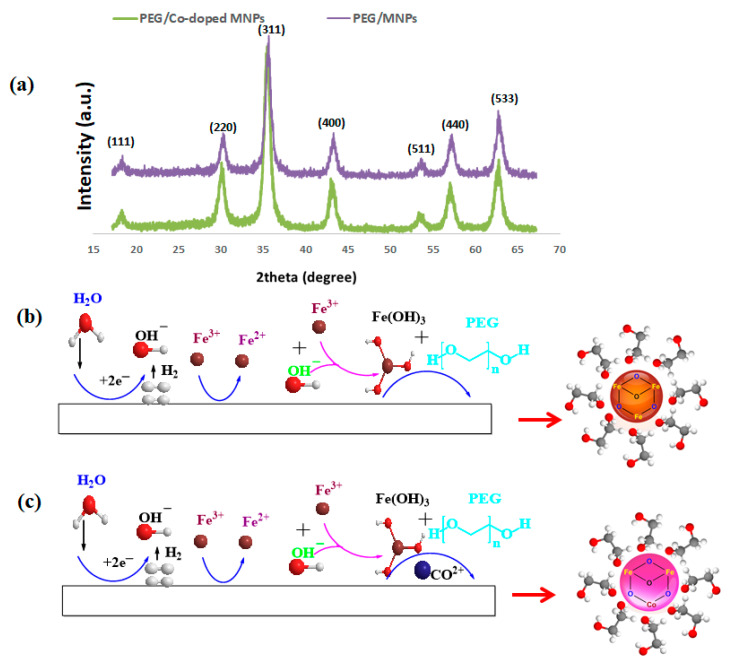
(**a**) XRD patterns of the PEG-MNPs and Co-doped PEG-MNPs; and (**b**,**c**) schematic mechanism of preparation of PEG-MNPs and Co-doped PEG-MNPs, respectively.

XRD patterns of the deposited magnetite samples are given in Figure 2a., where the diffraction planes of (111), (220), (311), (400), (422), (511), (440) and (533) are present at the 2θ values of 18.72°, 30.45°, 35.22°, 42.14°, 52.95°, 57.02°, 62.32° and 74.26°, respectively. These diffraction signatures suggest the formation of pure cubic crystalline Fe_3_O_4_ with JCPDS number of 01-074-1910 [20,21,22,31]. By the use of the Debye–Scherrer equation (D = Kλ/*β*cosθ) and based on diffraction plane of (311), the average size of crystallite zones (D) were 11.9 and 12.6 nm for the PEG-MNPs and Co^2+^-doped PEG-MNPs, respectively.

The ionic radii of Co^2+^ and Fe^2+^ cations (i.e., r ≈ 70 pm) are indicative of the fact that Co^2+^ doping into magnetite crustal structure should pose no obvious effect on the XRD pattern, as well as the crystal structure of the deposited iron oxide. It should be noted that magnetite has a cubic inverse spinel ferrite structure, in which Fe^3+^ atoms are only positioned in the octahedral sites, while both Fe^2+^ and Fe^3+^ cations can be found in the tetrahedral sites. The XRD results suggest that Mn^2+^ cations are incorporated into the magnetite crystal structure by occupying some sites related to the Fe^2+^ cations. This means that Co^2+^ cations act as Fe^2+^ cations during the cathodic deposition process with no privileged positioning [34]. The following mechanism can be used to explain the formation of such product.

Electrochemical step:

(1) $$2H_{2}O+2e^{-}\to 2OH^{-}+H_{2},$$

Chemical step:

(2) $$2Fe^{3+}+(1-x)Fe^{2+}+xCo^{2+}+5OH^{-}\to Fe^{3+}+Fe^{2+}+(1-x)Co_{x}O_{4}+1/2H_{2}O,$$

For the surface treatment, we added PEG to the electrodeposition solution, considering that PEG does not contribute to the electrochemical reaction occurring on the cathode electrode. Therefore, the above written reactions (Equations (1) and (2)) are likely to take place. After the nucleation and growth of Fe_3_O_4_ particles on the cathode electrode was completed, their surface was capped in-situ by the PEG layers, as schematically shown in Figure 2b,c.

**Figure 3:**
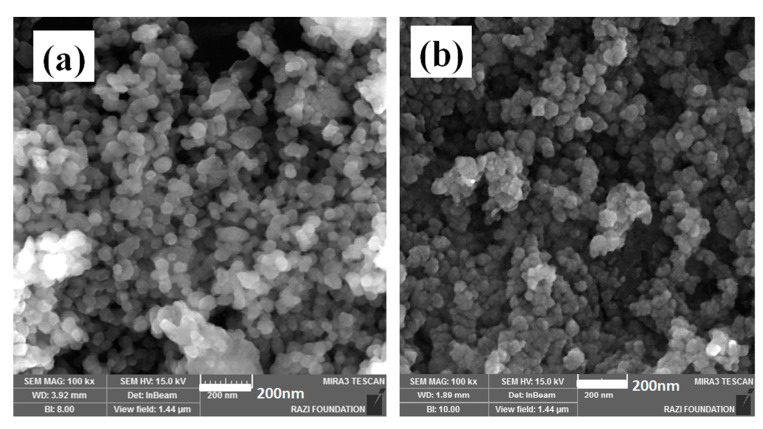
FE-SEM micrographs provided from the (**a**) PEG-MNPs and (**b**) Co^2+^-doped PEG-MNPs surfaces.

Surface morphology of MNPs was observed by FE-SEM (Figure 3), showing that spherical particles having an average diameter of 15–20 nm were achieved.

**Figure 4:**
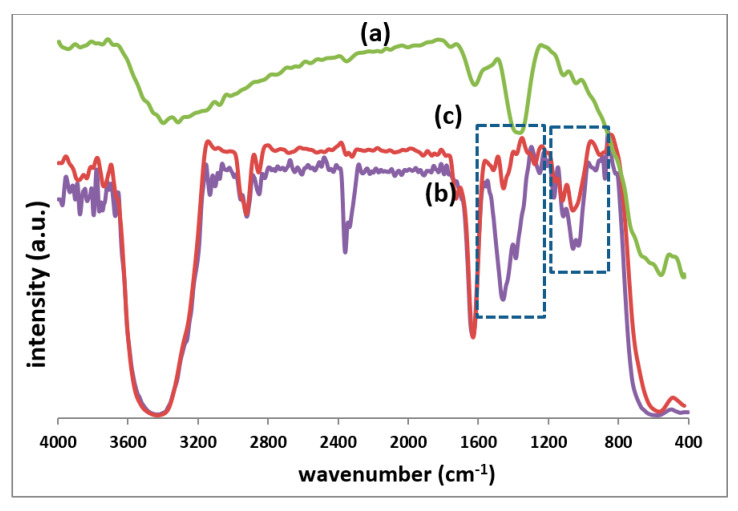
FTIR spectra of the fabricated: (**a**) bare MNPs; (**b**) PEG-MNPs; and (**c**) Co-doped PEG-MNPs.

Chemical structure changes were detected by FTIR technique (Figure 4). The unconditionally observed absorption peaks at about 432 and 592 cm^−1^ correspond to the stretching vibration modes of Fe^2+^–O–Co^2+^ and Fe^3+^–O–Fe^2+^ metallic bonds, respectively [32,33,35]. Moreover, some characteristic peaks were observed in two spectra assigned to PEG-functionalized particles (Figure 4a,b), in the interval of 3000 to 700 cm^−1^. Possible vibrations in this range are C-H stretching vibrations at 2926 and 2876 cm^−1^, CH_2_ vibrations at 1465 and 1399 cm^−1^, C–O–C stretching and bending vibrations at 1262 and 1168 cm^−1^, C–H wagging at 872 cm^−1^, C–C vibration at 1070 cm^−1^ and C–O–H stretching vibration at 1031 cm^−1^, which altogether prove of the formation of PEG macromolecular layer on the PEG-MNPs and Co^2+^-doped PEG-MNPs surface. The intensities of the band at 1465 cm^−1^ was increased by 66%, changing from 0.485 to 0.805, and for the band at 1399 cm^−1^ it was increased by 47% from 0.630 to 0.924 for the Co^2+^-doped PEG-MNPs with respect to the PEG-MNPs. Moreover, the rise in the intensity of the band at 1070 cm^−1^ was about 9% from 0.664 to 0.727. Such quantitative analyses are indicative of the higher activity of the surface in the case of Co^2+^-doped PEG-MNPs, i.e., the advantage of bulk modification.

**Figure 5:**
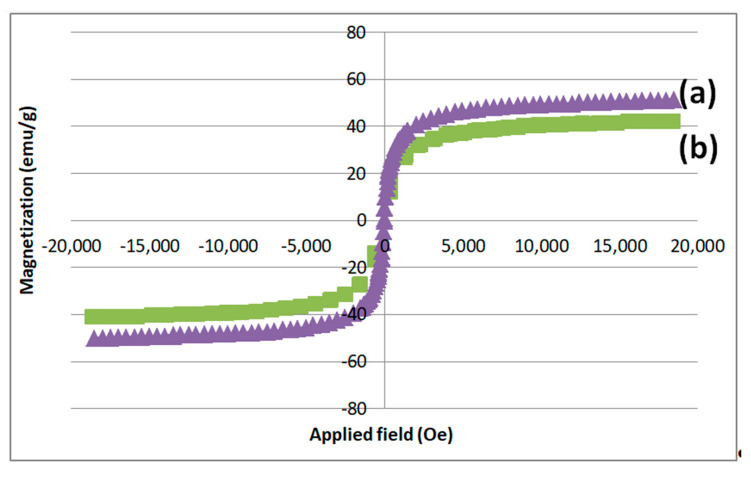
VSM plots of the synthesized: (**a**) PEG-MNPs; and (**b**) Co^2+^-doped PEG-MNPs.

Figure 5 shows the VSM plots obtained for the synthesized MNPs. The most important parameters including the saturation magnetization (Ms), coercivity (Ce) and the remanence (Mr) quantities were extracted and compared: for PEG-capped MNPs, Ms = 49.52 emu/g, Mr = 0.72 emu/g and Ce = 1.45 Oe, while, for Co^2+^-doped PEG-MNPs, Ms = 41.55 emu/g, Mr = 0.66 emu/g and Ce = 1.02 Oe. From these magnetic data, it was recognized that the Ms values of PEG-capped and Co-doped MNPs declined because of surface capping by non-magnetic organic layer and crystal structure doping with Co^2+^ cations; however, the superparamagnetic behavior of these samples improved to the observed reduction in Ce and Mr values.

**Figure 6:**
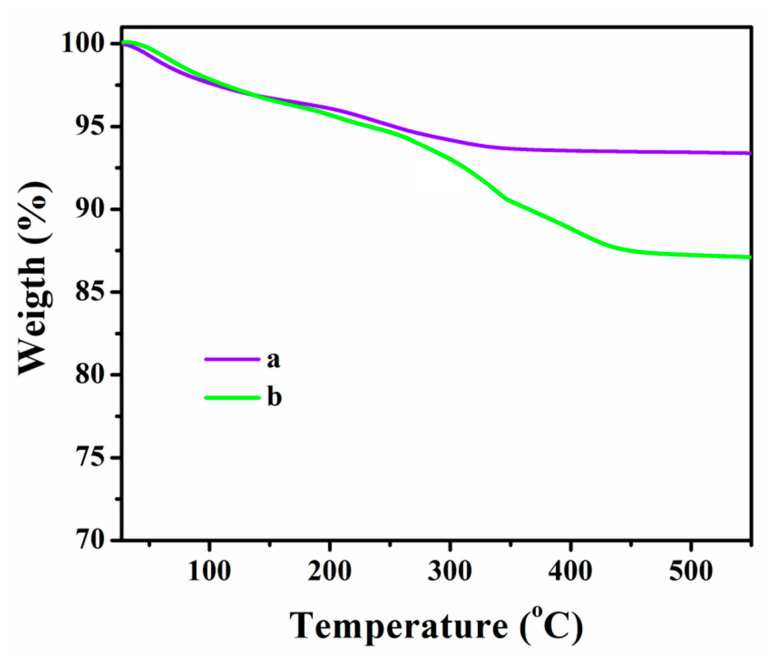
TGA data of: (**a**) PEG-MNPs; and (**b**) Co^2+^-doped PEG-MNPs.

Thermal decomposition behavior of the PEG-MNPs and Co^2+^-doped PEG-MNPs are compared in Figure 6. The early degradation behavior around 100 °C can be attributed to moisture and small molecules attached on the surface of nanoparticles. The main degradation in the temperature range of 200–450 °C can be related to degradation of PEG on the surface of nanoparticles. At the end of TGA experiment (at 550 °C), PEG-MNPs exhibited a weight loss of about 6.7%, while the weight loss values calculated for Co^2+^-doped PEG-MNPs was about 12.9%. This difference (6.2%) could arise from the higher number of PEG groups anchored to the surface of Co^2+^-doped MNPs due to higher reactivity of Fe_3_O_4_ surface in the presence of Co.

### 3.2. Analysis of Cure Reaction

**Figure 7:**
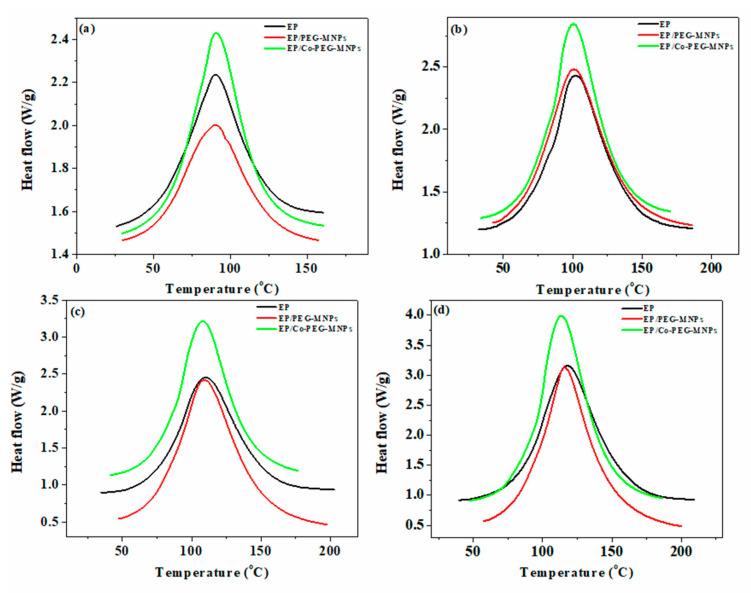
DSC thermograms of EP, EP/PEG-MNPs and EP/PEG-Co-doped MNPs obtained nonisothermally carrying the heating rate (**a**) 5 °C·min^−1^, (**b**) 10 °C·min^−1^, (**c**) 15 °C·min^−1^ and (**d**) 20 °C·min^−1^

Crosslinking reactions in thermosetting systems strongly affect ultimate properties [36,37,38]. DSC thermograms of neat epoxy and its nanocomposites containing 0.1 wt.% of PEG modified-MNPs and -Co^2+^-doped MNPs were obtained nonisothermally at different heating rates (*β*) of 5, 10, 15 and 20 °C min^−1^ (Figure 7). The unimodal exothermic peaks unconditionally observed in DSC thermograms of all samples at all heating rates are indicative of the single-step kinetics of epoxy ring opening [39,40]. The crosslinking reaction of the studied epoxy nanocomposites passes through different reactions, including the primary and secondary amines of curing agent and the etherification reaction caused by hydroxyl groups formed during the epoxide ring opening reaction as well as OH groups of PEG on the surface of MNPs [41,42]. The typical reactions taking place between PEG-modified and PEG-Co^2+^-doped MNPs and the resin springs from the hydroxyl groups of surface functionality (PEG) with epoxy along with amine groups of curing agent. At the beginning of the curing reaction, the hydrogen of primary amine of curing agent reacts with the oxirane rings of epoxy resin that forms a secondary amine. Then, the hydrogen of the secondary amine with less reactivity and accessibility participates in epoxide ring opening and forms nonreactive tertiary amine. At the late stage of curing reaction, the hydroxyl groups formed during the reaction of epoxy groups with amine groups of curing agent and OH groups of PEG on the surface of nanoparticles having less reactivity can participate in the epoxide ring via etherification reaction [43].

In recent studies, we used *Cure Index* as a fast-detecting criterion to classify nanocomposites in terms of their crosslinking ability with respect to the neat resin, which can be calculated as *Cure Index* = ΔT* × ΔH*. The dimensionless enthalpy is defined as ΔH* = ΔH_Comp._/ΔH_Ref__._, where the numerator and denominator are the enthalpy of cure of the composite and that of neat resin (reference or control sample), respectively. Likewise, dimensionless cure window is defined as ΔT* = ΔT_Comp_/ΔT_Ref__._), where *ΔT = T_endset_ – T_onset_* and the numerator and the denominator are the ΔT of the composite and that of neat resin, respectively [44,45]. Such quantities together with the exothermal peak temperature (*T_p_*) and the total heat released during crosslinking reaction (*ΔH*) as a function of heating rate applied in DSC test are included in Table 1.

**Figure 8:**
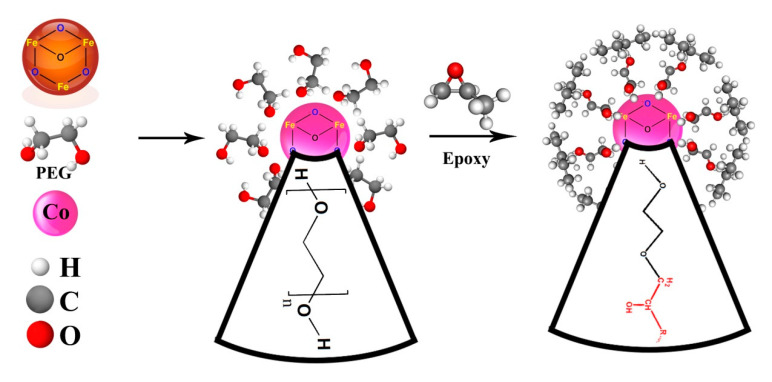
Possible reaction taking place between the PEG-Co-doped MNPs and the epoxy resin.

From a molecular view, at the early stage of curing reaction, the presence of PEG-MNPs or Co-doped PEG-MNPs in the epoxy resin makes the curing reaction slow because of enhanced interaction between particles and cure; therefore, the viscosity of the system increases. Such a hindered cure reaction was detected and proved by the increase in the value of *T_onset_* of the systems containing MNPs. However, at an intermediate stage of crosslinking, when the gelation is likely to take place, the presence of PEG-MNPs and Co-doped PEG-MNPs with hydroxyl groups on their surfaces accelerates the curing reaction, as featured by a fall in the value of *T_p_* of nanocomposites compared to that of the neat epoxy. Table 2 shows the increase in the values of *ΔH* of the epoxy nanocomposites due to the participation of OH groups of PEG in epoxide ring opening [38,46]. This reaction is schematically illustrated in Figure 8. The long arms of PEG on the surface of MNPs diffuse through the crosslinked networks of epoxy at later stages of cure and react with the remainder of epoxide rings, which results in accelerating the curing when gelation is dominant (diffusion-controlled curing). This effect became more significant in the vicinity of the interfacial surface area of nanoparticles and epoxy resin. Therefore, the nanoparticles played the role of curing promoter after vitrification where the reaction was under the control of diffusion. The MNPs and Co-doped MNPs functionalized with huge PEG molecules diffused into the dense crosslinked network and resulted in higher *ΔH* values in comparison with that of the neat epoxy. In the case of PEG-Co-doped MNPs, due to the higher amount of PEG that anchored on the surface of nanoparticle, higher *ΔH* values were obtained compared to the EP/PEG-MNPs.

The values of ΔT* and ΔH* are lower for the EP/PEG-Co-doped MNPs compared to the corresponding values obtained for the EP/PEG-MNPs system. As discussed above, Co^2+^ dopant prefers to locate in the bulk layers of MNPs; therefore, the ions on the surface of lattice give the Fe_3_O_4_ surface a reactive nature [47]. From this perspective, probably more PEG macromolecules had to be adsorbed on the surface of Co-doped MNPs—a possible reason for the epoxide ring opening by the OH groups of PEG compared to the epoxy and PEG-MNPs. Evidently, the former has higher ΔH* values at all heating rates compared to the latter. At the low heating rate, the *Cure Index* values for the EP/PEG-MNPs nanocomposite shows *Poor* cure state, possibly because the cuing moieties had enough time to participate in curing reaction but possessed less kinetic energy per molecule available for PEG-coated MNP to participate in epoxy curing reaction [48,49]. However, at higher heating rates, the EP/PEG-MNPs nanocomposite was marked as *Excellent* as both the time and energy were adequate for curing moieties. Regardless of the heating rate, Co-doped PEG-MNP participated in epoxy curing reaction more effectively and took the label of *Excellent* [49,50].

The conversion of the curing reaction, α, was calculated by using the following equation:

(3) $$\alpha =\frac{\Delta H_{T}}{\Delta H_{\infty }},$$

**Figure 9:**
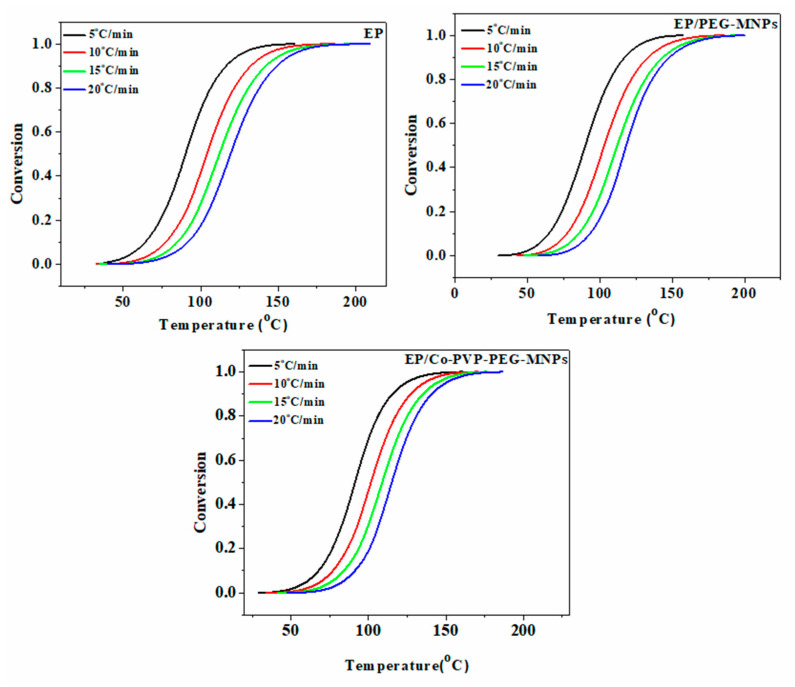
The variation of α in terms of reaction temperature for the neat resin, EP (the reference sample) and EP/PEG-MNPs and EP/Co-PEG-MNPs nanocomposites as a function of heating rate (5, 10, 15 and 20 °C·min^−1^).

In Equation (3), the *ΔH_∞_* and *ΔH_T_* parameters are the total enthalpy of the complete cure reaction and the heat release up to a specific temperature T, respectively. The variation of α by the curing temperature was calculated for the neat resin [51,52] as well as for the epoxy nanocomposites as a function of heating rate (Figure 9). The S-shape *α–T* curves unconditionally obtained for all studied systems are a signature of the autocatalytic nature of curing reaction. It means that the curing reaction is initiated by the reaction of epoxy with amine groups of the curing agent until the gelation takes place. At higher temperatures, the hydroxyl groups belonging to the PEG and those formed in the course of ring opening of epoxy participate in curing reaction through etherification reaction. As a final point, the curing rate is slowed down by the occurrence of vitrification.

The rate of cure was calculated as:

(4) $$\frac{d\alpha }{dt}=k(T)f(\alpha ),$$

where *f(α)* is representative of the reaction model as a function of α and *k(T)* is the reaction rate constant according to the Arrhenius equation:

(5) $$k(T)=Aexp(-\frac{E_{\alpha }}{RT}),$$

In Equation (5), *A*, *R* and *E_α_* are the pre-exponential factor, the universal gas constant and the activation energy of curing reaction, respectively. By substituting *k(T)* from Equation (5) into Equation (4), the rate of curing reaction is yielded:

(6) $$\frac{d\alpha }{dt}=Aexp(-\frac{E_{\alpha }}{RT})f(\alpha ),$$

To estimating the reaction rate, the values of activation energy should be obtained. For this purpose, model-free isoconversional methods were used to calculate *E_α_* as a function of temperature in a given *α* [53]. The well-known *Friedman* and the Kissinger–Akahira–Sunose (*KAS*) isoconversional methods were used, represented in Equations (7) and (8), respectively.

(7) $$ln\left[\beta_{i}{\left(\frac{d\alpha }{dT}\right)}_{\alpha ,i}\right]=ln\left[f(\alpha )A_{\alpha }\right]-\frac{E_{\alpha }}{RT_{\alpha ,i}},$$

(8) $$ln\;\left(\frac{\beta_{i}}{T_{\alpha ,i}^{2}}\right)=Const-1.0008\;\;\;\left(\frac{E_{\alpha }}{RT_{\alpha }}\right),$$

**Figure A1:**
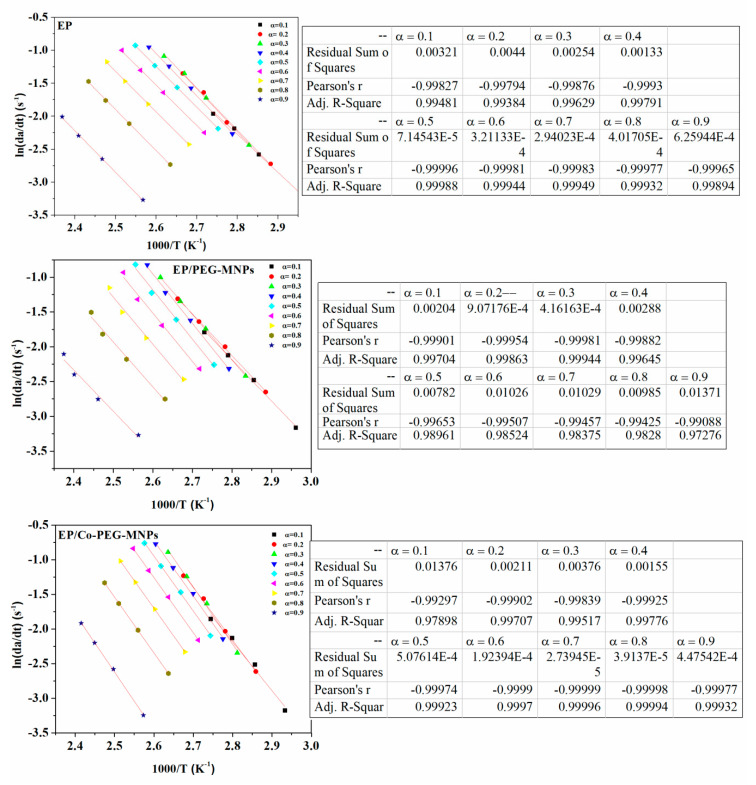
Arrays of *ln*(*dα/dt*) plots vs. 1/T as typical plots obtained for the studied samples using Friedman model at *β* = 5 °C·min^−1^.

**Figure A2:**
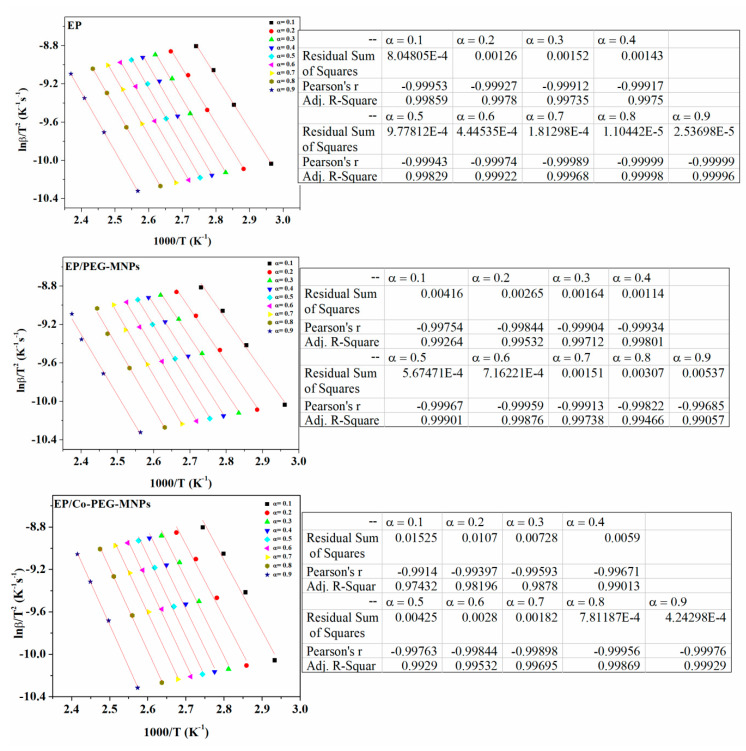
Arrays of *ln*(*β*/T^2^) plots vs. 1/T as typical plots obtained for the studied samples using *KAS* model.

The value of *E_α_* for a given *α* is obtained from the slope of the curve of $ln\;\;\left[\beta_{i}{\left(d\alpha /dT\right)}_{\alpha ,i}\right]$ vs. 1/*T_α_* (Equation (7)) and $ln\;\;\left(\beta_{i}/T_{\alpha ,i}^{2}\right)$ vs. 1/T (Equation (8)), plotted in Figure A1 and Figure A2 in Appendix A, respectively.

**Figure 10:**
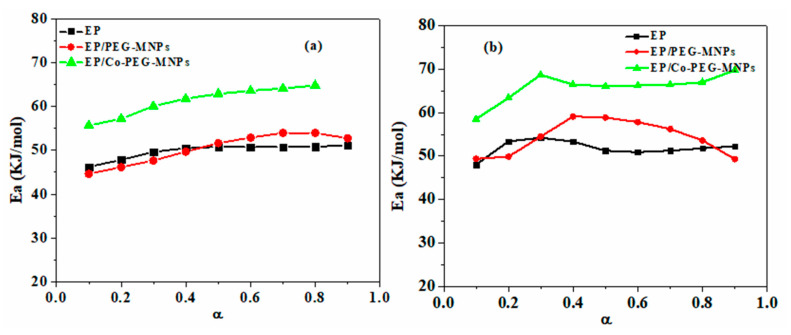
The variation of *E_α_* for EP, EP/PEG-MNPs and EP/Co-PEG-MNPs nanocomposites according to: (**a**) the *Friedman* model; and (**b**) the *KAS* model.

Figure 10 shows the variation of *E_α_* as a function of *α* obtained by the *Friedman* and *KAS* models for the studied systems. The outcomes of the two methods are expectedly similar, where the *E_α_* plot follows an ascending trend for possible autocatalytic reactions taking place at later stages of cure. Moreover, a shift to higher *E_α_* is obvious for systems containing MNPs, particularly for the bulk surface-functionalized MNPs, due to the intensified epoxy ring opening [54]. The curing reaction of epoxy system is progressed through chemically crosslinking reaction, but, after the occurrence of vitrification, it went through the diffusion-controlled mechanism [43].

For EP/Co-PEG-MNPs nanocomposites, participation of the hydroxyl groups of the PEG in cure reaction accelerated the crosslinking by increasing the possibility of reaction at higher conversions [55]. The population of reactive hydroxyls assisted in cure reaction continuation once gelation and vitrification in the EP/Co-PEG-MNPs system were initiated. Thus, a significantly higher value of *E_α_* of the assigned system with respect to the reference resin was the result of excessive interaction between epoxide and curing agents.

**Figure A3:**
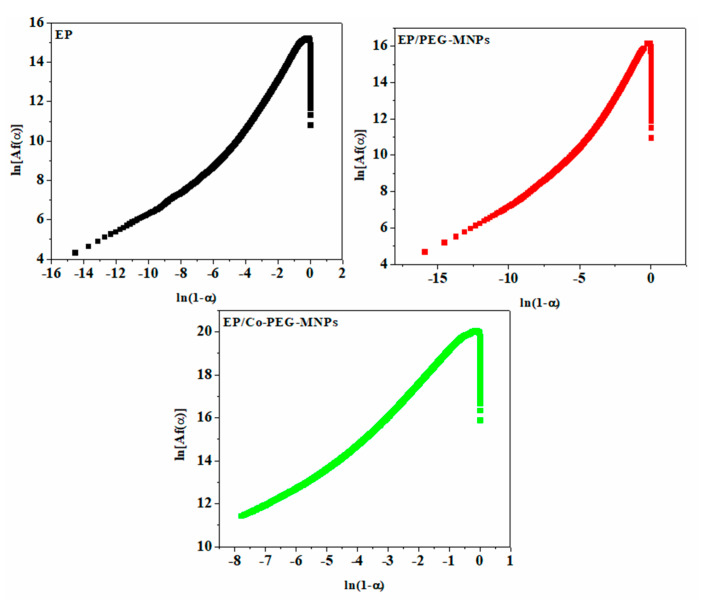
Arrays of *ln [Af(α)]* plots vs. *ln*(1-*α*) as typical plots obtained for the studied samples using *Friedman* model.

In the next step, to determine the reaction model, we used the *Friedman* and *Malek* methods. The details of *Friedman* method and its mathematical relation can be found in Appendix B. The curing mechanism in the system can be estimated according to Equation (A1) by looking at the shape of the plot of *ln[Af(α)]* vs. *ln*(1-*α*). Crosslinking reaction of the neat epoxy and two nanocomposites progressed autocatalytically, as the maximum points derived from the results in Figure A3 are placed in the range of 0.2 < *α* < 0.4.

A more accurate *Malek* method was also used for determination of kinetic model by considering the maximum points of Malek parameters of *y(α) = (α_m_), z(α) = (α_p_^∞^)*, which are defined in Equations (9) and (10), respectively, and the conversion at the maximum point of DSC curves (*α_p_*).

(9) $$y(\alpha )={\left(\frac{d\alpha }{dt}\right)}_{\alpha }exp\left(\frac{E_{0}}{RT_{\alpha }}\right)=Af(\alpha ),$$

(10) $$z(\alpha )={\left(\frac{d\alpha }{dt}\right)}_{\alpha }T_{\alpha }^{2},$$

**Figure 11:**
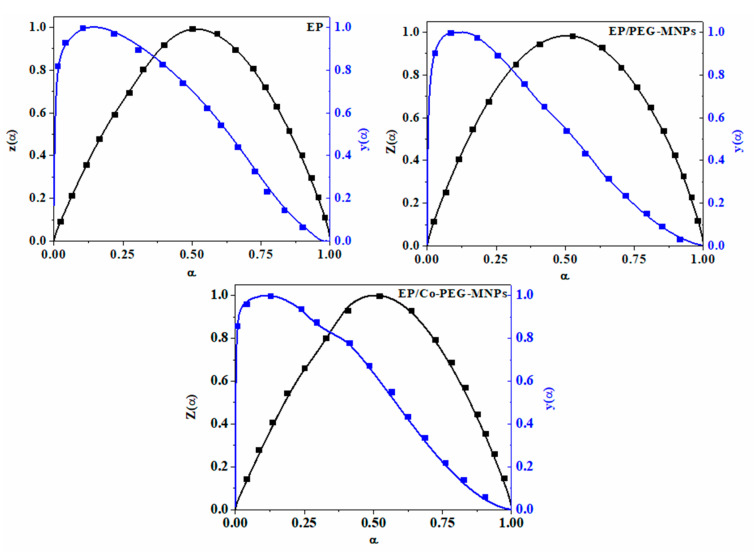
The shape and alteration pattern of *y(α)* and *z(α)* versus the extent of reaction captured by the Malek model.

The values of *y(α)* and *z(α)* are normalized with respect to their maximum values to take values between 0 and 1 (see Figure 11) [56,57]. The values of *α_m_*, *α_p_* and *α_p_^∞^* for the prepared samples are summarized in Table 2.

By comparing Figure 11 with *Malek* master plots and according to the data in Table 2 (which indicate that *α_m_* is lower than *α_p_* and *α_p_^∞^* < 0.632), it was concluded that a two-parameter autocatalytic kinetic model can be considered for EP, EP/PEG-MNPs and EP/Co-PEG-MNPs.

Two-parameter autocatalytic kinetic model was defined by Sestak–Berggren as follows:

(11) $$f(\alpha )=\alpha^{m}{\left(1-\alpha \right)}^{n},$$

where *n* and *m* are non-catalytic and autocatalytic reaction orders, respectively. The values of the orders of reactions (*n* and *m*) as well as the pre-exponential factor (*lnA*) are determined from Equations (A2) and (A3) found in Appendix C and listed in Table 3. In addition, the average value of *E_α_* (*Ē_α_*) from *Friedman* and *KAS* methods is calculated, as given in Table 3.

From the data in Table 3, it is apparent that both the isoconversional approaches predicted similar trends and values. The cumulative order of crosslinking reaction (*m*+*n*) took a value higher than one, demonstrating the complexity of the curing reaction in the studied systems [58].

The presence of both PEG-MNPs and Co-PEG-MNPs nanoparticles in the epoxy matrix increased the reaction rate compared to the neat epoxy. Moreover, once PEG-MNPs and Co-PEG-MNPs were incorporated into the epoxy, the interaction between reactive groups with the curing moieties in the epoxy system increased the possibility of collision in the system, as reflected in higher values of *ln(A)*, and consequently higher *E_α_* values were obtained (Collison in Arrhenius principle is defined as the number of contacts between molecules per unit volume). Since viscosity effect is dominantly acting against curing in such systems, more collisions would be liable for an improved curing at later stages of cure reaction.

To obtain the kinetics parameters for the studied rate of cure reaction (*dα/dt*), it is calculated as:

(12) $$\frac{d\alpha }{dt}=Aexp(-\frac{E_{\alpha }}{RT})\alpha^{m}{\left(1-\alpha \right)}^{n},$$

**Figure 12:**
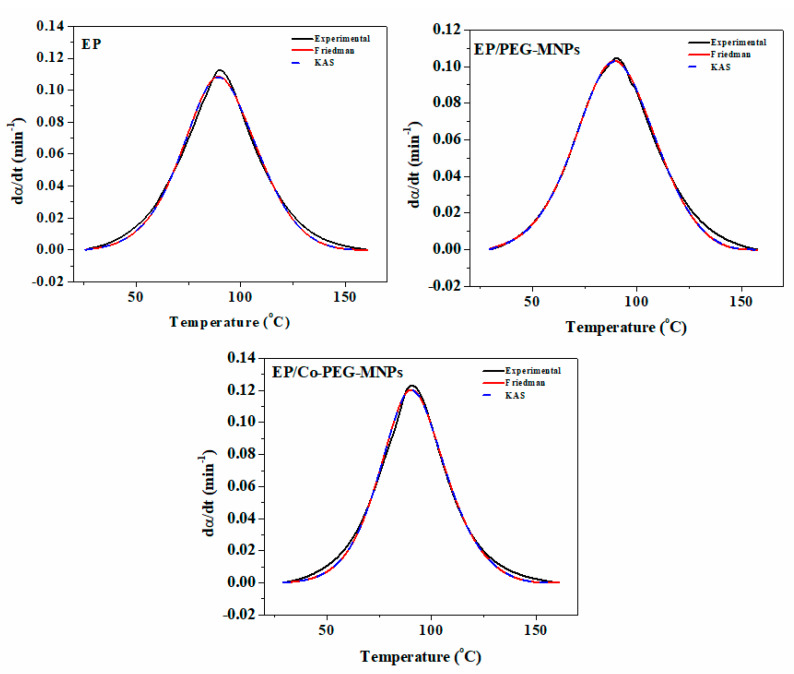
The experimental and the calculated variation of the conversion of the prepared samples (rate of cure reaction) as a function of time.

Figure 12 compares the *dα/dt* obtained from Equation (12) with the experimental values. Fortunately, the predicted *dα/dt* appropriately fitted the experimental data.

### 3.3. Glass Transition Analysis

The values of *T_g_* of the EP, EP/PEG-MNPs and EP/Co-PEG-MNPs were obtained in the reheating cycle from 20 to 250 °C at *β* of 10 °C·min^−1^ for the samples, and the results are reported in Table 4.

The *T_g_* of epoxy was slightly increased by addition of 0.1 wt.% of PEG-MNPs, suggesting enhanced interaction between the PEG functional groups on the surface of MNPs and the curing moieties. In comparison with neat epoxy, addition of 0.1 wt.% Co-PEG-MNPs resulted in increase of *T_g_* from 101 to 105 °C. This rise in *T_g_* of EP/Co-PEG-MNPs nanocomposite is due to the increase in the interfacial area between the Co-PEG-MNPs and epoxy leading to higher crosslinking density, as apparent from the higher *ΔH* value of this system. It can be realized that the presence of Co-PEG-MNPs with higher amount of PEG on its surface compared to that of MNPs caused hindrance of segmental mobility of epoxy chains due to the formation of denser epoxy crosslinked network, in which more active sites are present on the surface of Co-PEG-MNPs for reacting with epoxide group [59].

## 4. Conclusions

Surface-bulk modification of nanoparticles was applied in making highly-crosslinked epoxy nanocomposites. The galvanostatic cathodic deposition method was applied in synthesis of the PEG capped-MNPs and Co-doped MNPs as model nanoparticles. FTIR, XRD, FESEM, VSM and TGA analyses were performed to confirm positioning of elements and molecules at the surface and in the bulk of particles. FESEM micrographs proved spherical particles formed having an average diameter of 15–20 nm. The FTIR bands proved the presence of PEG on the surface of both MNPs and Co-doped MNPs. Epoxy nanocomposites containing 0.1 wt.% PEG-coated MNPs and Co-doped MNPs were then prepared and studied by nonisothermal DSC for evaluating their curing potential. Co dopant mainly positioned in the bulk layers of MNPs, which led to change in the reactivity of the surface such that more PEG was anchored on the surface of Co-doped MNPs. It was proved by TGA data that Co-doped MNPs have 6.2% more PEG on their surface, which resulted in increased ΔH* values. The nanocomposites cured at all heating rates took *Excellent* cure label according to the *Cure Index*. Both *Friedman* and *KAS* isoconversional methods proved higher *E_α_* for the epoxy system in the presence of Co-PEG-MNPs nanoparticles. Well-achieved confidence between the experimental rate of cure reaction and the predicted values from the *Friedman* and *KAS* methods denoted the appropriateness of these models. The *T_g_* data indicate that presence of Co-PEG-MNPs in epoxy matrix with higher amount of PEG on its surface compared to MNPs caused hindrance of segmental mobility of epoxy chains, which featured an increase in the *T_g_* value from 102 to 105 °C due to the formation of denser epoxy crosslinked network. This approach can be applied to develop multifunctional polymer composites in view of the fact that surface and bulk characteristics can affect network formation as well as the properties of polymer composites in different ways.

## Figures and Tables

**Table 1:** Main characteristics of cure reaction of the prepared epoxy nanocomposites in terms of heating rate.

Designation	*β* (°C·min^−1^)	*T_onset_* (°C)	*T_endset_* (°C)	*T_p_* (°C)	*ΔT* (°C)	*ΔH*_∞_ (J/g)	ΔT^*^	ΔH^*^	*Cure Index*	Quality
**EP**	5	25.82	160.63	90.223	134.81	361.04	n.a.	n.a.	n.a.	n.a.
	10	32.46	186.62	101.02	154.17	350.64	n.a.	n.a.	n.a.	n.a.
	15	34.64	202.64	109.94	168	314.54	n.a.	n.a.	n.a.	n.a.
	20	39.93	209.27	118.03	169.34	336.89	n.a.	n.a.	n.a.	n.a.
**EP/PEG-MNPs**	5	29.61	157.45	90.173	127.84	308.13	0.95	0.85	0.81	*Poor*
	10	42.66	186	100.88	143.34	368.31	0.93	1.05	0.98	*Excellent*
	15	47.43	197.45	109.12	150.03	384.11	0.89	1.22	1.09	*Excellent*
	20	57.55	199.92	115.88	142.36	346.27	0.84	1.03	0.86	*Excellent*
**EP/Co-PEG- MNPs**	5	29.12	160.86	90.677	131.74	446.35	0.98	1.24	1.21	*Excellent*
	10	33.91	170.41	100.49	136.50	396.33	0.89	1.13	1.00	*Excellent*
	15	41.39	176.38	107.98	135.00	365.32	0.80	1.16	0.93	*Excellent*
	20	47.56	186.56	113.62	139.00	389.25	0.82	1.16	0.95	*Excellent*

n.a., not applicable (reference measurements).

**Table 2:** The quantities of *α_p_, α_m_* and *α_p_^∞^* due to Malek model as a function of heating rate.

Designation	Heating Rate (°C·min^−1^)	*α_p_^∞^*	*α_m_*	*α_p_*
**EP**	5	0.501	0.099	0.517
	10	0.403	0.127	0.505
	15	0.413	0.228	0.484
	20	0.451	0.208	0.501
**EP/PEG-MNPs**	5	0.505	0.126	0.527
	10	0.333	0.113	0.485
	15	0.345	0.096	0.470
	20	0.341	0.113	0.467
**EP/Co-PEG-MNPs**	5	0.484	0.113	0.508
	10	0.550	0.076	0.495
	15	0.567	0.233	0.505
	20	0.510	0.249	0.488

**Table 3:** The kinetic parameters obtained at different heating rates using *Friedman* and *KAS* models.

***Friedman***								
**Designation**	**Heating Rate (°C·min^−1^)**	**Ē_α_ (kJ/mol)**	**ln(A) (1/s)**	**Mean (1/s)**	**m**	**Mean**	**n**	**Mean**
**EP**	5	51.83	16.24	16.29	0.381	0.408	1.509	1.534
	10		16.30		0.421		1.545	
	15		16.28		0.383		1.539	
	20		16.32		0.447		1.543	
**EP/PEG-MNPs**	5	54.30	16.93	17.14	0.256	0.374	1.499	1.630
	10		17.10		0.335		1.637	
	15		17.13		0.379		1.664	
	20		17.39		0.528		1.719	
**EP/Co- PEG-MNPs**	5	65.86	21.01	21.05	0.354	0.380	1.661	1.662
	10		21.02		0.341		1.648	
	15		21.03		0.360		1.652	
	20		21.13		0.464		1.687	
***KAS***								
**Designation**	**Heating Rate (°C·min^−1^)**	**Ē_α_ (kJ/mol)**	**ln(A) (1/s)**	**Mean (1/s)**	**m**	**Mean**	**n**	**Mean**
**EP**	5	49.82	15.58	15.65	0.405	0.431	1.490	1.515
	10		15.67		0.444		1.526	
	15		15.65		0.405		1.519	
	20		15.71		0.469		1.524	
**EP/PEG-MNPs**	5	50.37	15.63	15.89	0.303	0.416	1.459	1.589
	10		15.85		0.377		1.594	
	15		15.90		0.420		1.622	
	20		16.18		0.564		1.679	
**EP/Co- PEG-MNPs**	5	61.82	19.68	19.76	0.397	0.421	1.626	1.627
	10		19.73		0.384		1.612	
	15		19.76		0.401		1.617	
	20		19.87		0.502		1.653	

**Table 4:** *T_g_* of fully cured neat epoxy and its nanocomposites at *β* of 10 °C min^−1^.

Sample	*T_g_* (°C)
**EP**	101.1
**EP/PEG-MNPs**	102.6
**EP/Co-PEG-MNPs**	105.3

**Figure A4:**
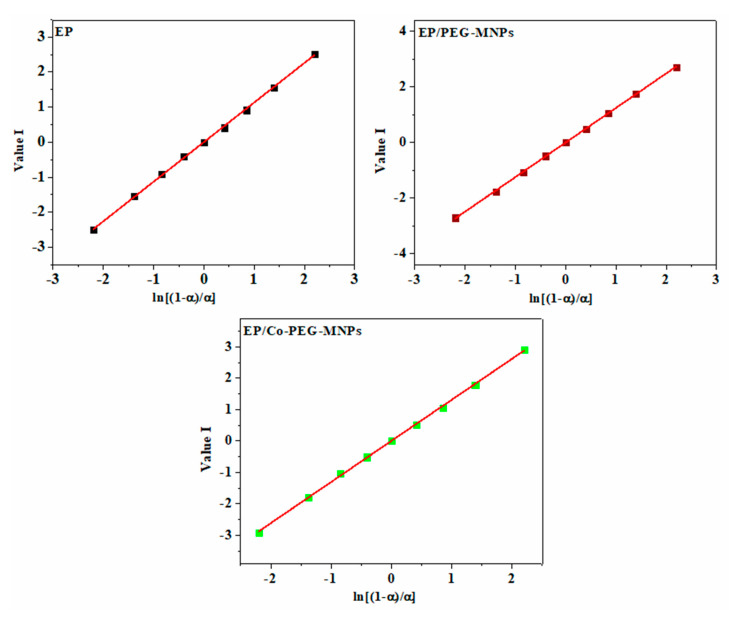
Variation of Value I of the samples prepared in this work at typical heating rate of 5 °C·min^−1^.

**Figure A5:**
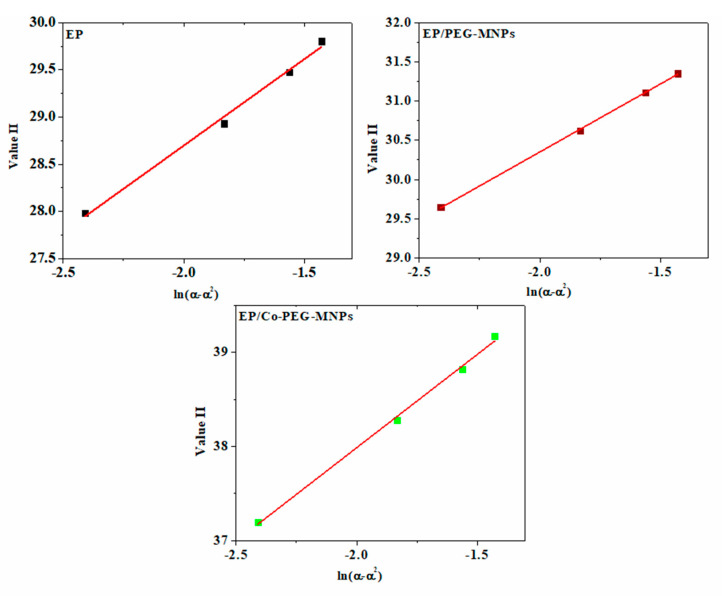
Variation of Value II of the samples prepared in this work at typical heating rate of 5 °C·min^−1^.

## Appendix A. Isoconversional Kinetic Methods

### Appendix A.1. Friedman Model

By plotting $ln\left[\beta_{i}{\left(d\alpha /dT\right)}_{\alpha ,i}\right]$ vs. *1/T_α_* from Equation (7), the value of *E_α_* is obtained from the slope of Figure A1.

### Appendix A.2. KAS Method

Plotting $ln\left(\beta_{i}/T_{\alpha ,i}^{2}\right)$ vs. 1/*T_α_* from Equation (8) for each *α* value gives a value for *E_α_* (Figure A2).

## Appendix B. Selection of Curing Reaction Model

### Friedman Model

This method can be applied on experimental data using Equation (A1). The shape of the plot of *ln*[*Af(α)*] vs. *ln*(1-*α*) can be used to determine whether or not the cure reaction has roots in autocatalytic reaction mechanism (Figure A3).

(A1) $$ln\left[Af(\alpha )\right]=ln\left(\frac{d\alpha }{dt}\right)+\frac{E}{RT}=lnA+nln\left(1-\alpha \right),$$

## Appendix C. Determination of Degree of Reaction

The set of Equations (A2) and (A3) should be solved simultaneously to explore the triplet parameters of cure, i.e., (*n*, *m*, *ln A*):

(A2) $$ValueΙ=ln\left(\frac{d\alpha }{dt}\right)+\frac{E_{\alpha }}{RT}-ln\left[\frac{d(1-\alpha )}{dt}\right]-\frac{E_{\alpha }}{RT\prime }=(n-m)ln\left(\frac{1-\alpha }{\alpha }\right),$$

(A3) $$ValueIΙ=ln\left(\frac{d\alpha }{dt}\right)+\frac{E_{\alpha }}{RT}+ln\left[\frac{d(1-\alpha )}{dt}\right]+\frac{E_{\alpha }}{RT\prime }=(n+m)ln(\alpha -\alpha^{2})+2lnA,$$
